# Hearing Aid Use Trends Among Older Adults by Income and Metropolitan vs Nonmetropolitan Residence

**DOI:** 10.1001/jamanetworkopen.2024.36140

**Published:** 2024-09-27

**Authors:** Sarah Y. Bessen, Wuyang Zhang, Emmanuel Garcia-Morales, Jennifer A. Deal, Nicholas S. Reed

**Affiliations:** 1 Department of Otolaryngology-Head and Neck Surgery, Johns Hopkins University, Baltimore, Maryland; 2 Department of Epidemiology, Johns Hopkins Bloomberg School of Public Health, Baltimore, Maryland; 3 Cochlear Center for Hearing and Public Health, Department of Epidemiology, Johns Hopkins Bloomberg School of Public Health, Baltimore, Maryland; 4 Optimal Aging Institute, NYU Grossman School of Medicine, New York, New York

## Abstract

This cross-sectional study assesses the prevalence of hearing aid use among older US adults with hearing loss and stratifies the findings by income and metropolitan vs nonmetropolitan residence.

## Introduction

Hearing loss is highly prevalent in the US and is associated with adverse aging outcomes.^1^ However, fewer than 20% of US adults with hearing loss use hearing aids, the mainstay of treatment, due to limitations in access and quality.^1^ The effects of geographic location and income on hearing aid use over time are not known.

## Methods

The population of this cross-sectional study included community-dwelling participants from the 2011, 2015, and 2022 cycles of the National Health & Aging Trends Study (NHATS), an annual cohort study of US adults 65 years and older.^2^ Initiated in 2011, NHATS replenished its sample in 2015 and 2022 to maintain national representativeness. Participants were asked if they had used a hearing aid or other hearing device in the last month. Annual household income was categorized as low (below the federal poverty line [FPL]), middle (100%-200% of the FPL), or high (≥200% of the FPL). Residence in a metropolitan vs nonmetropolitan county was derived by linking each participant’s county of residence to the 2013 Rural-Urban Continuum codes.^3^

We applied NHATS complex analytical weights to all analyses to account for sampling design and national representativeness of the data.^4^ Statistical analyses were conducted using Stata, version 18.0 (StataCorp LLC).

The Johns Hopkins Bloomberg School of Public Health Instutional Review Board approved NHATS. Participants provided informed consent at the time of enrollment. Data used in the present study are publicly available and deidentified. We followed the STROBE reporting guideline.

## Results

A total of 7089, 6885, and 5459 participants were included from the NHATS cycles for 2011, 2015, and 2022, respectively. The proportion of older adults reporting hearing aid use increased from 11.2% (95% CI, 10.3-12.0) in 2011 to 16.3% (95% CI, 14.8-18.0) in 2022, a 45.5% increase in uptake (Table). While the overall proportion of older adults with hearing aid use was higher in nonmetropolitan vs metropolitan areas, more growth in hearing aid use was observed among participants in metropolitan areas and among participants with higher income irrespective of residence (Table). Hearing aid use increased across all groups except for low-income adults in nonmetropolitan areas, for whom there was a 15.2% decrease (Table, Figure).

**Table.** Weighted Proportions of US Adults 65 Years and Older Using Hearing Aids, by Residence and Income, in the National Health and Aging Trends Study, 2011-2022^a^

	Study cycle year			% Change
	2011 (n = 7089)	2015 (n = 6885)	2022 (n = 5459)	
Total	11.2 (10.3-12.0)	13.3 (12.4-14.3)	16.3 (14.8-18.0)	45.5
Metropolitan residence	10.7 (9.8-11.8)	13 (12.0-14.1)	16.1 (14.5-17.8)	50.5
Low income	8.7 (6.5-11.6)	7.4 (5.4-10.0)	9.3 (6.3-13.6)	6.9
Middle income	9.6 (8.4-11.0)	10.3 (8.8-11.9)	13.8 (10.7-17.5)	43.8
High income	11.6 (10.4-12.9)	14.8 (13.5-16.2)	17.9 (15.9-19.9)	54.3
Nonmetropolitan residence	13.1 (11.7-14.7)	14.8 (12.4-17.6)	17.6 (14.1-21.8)	34.4
Low income	11.2 (8.6-14.6)	12.4 (6.3-23.0)	9.5 (2.9-27.0)	−15.2
Middle income	11.6 (8.1-16.5)	15.6 (11.1-21.4)	11.7 (7.2-18.5)	0.9
High income	14.4 (12.4-16.6)	15 (12.5-17.8)	21.5 (16.4-27.8)	49.3

^a^ Data are presented as the percentage (95% CI) of participants. Low income was defined as below the federal poverty line (FPL); middle income, as 100%-200% of the FPL; and high income, as greater than 200% of the FPL.

**Figure.**
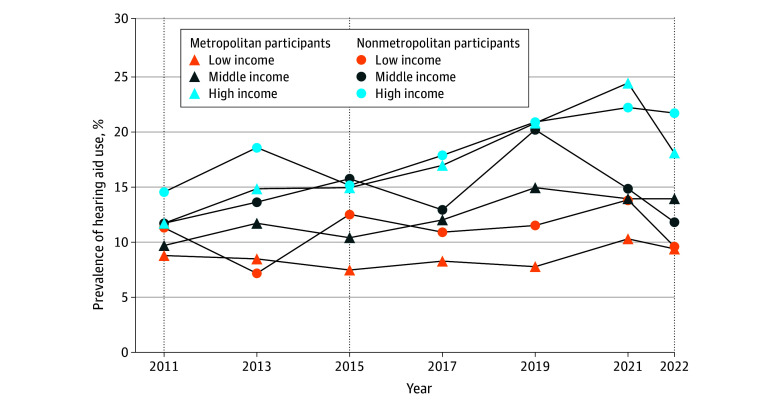
Prevalence of Hearing Aid Use Among US Adults 65 Years and Older in the National Health and Aging Trends Study, 2011 to 2022 Prevalence estimates from 2011, 2015, and 2022 (dashed vertical lines) include a nationally representative sample.

## Discussion

In this nationally representative cross-sectional study, the proportion of adults aged 65 years and older reporting hearing aid use increased by 45.5% between 2011 and 2022, with the greatest increase occurring among adults who resided in metropolitan areas. Notably, hearing aid use decreased among adults with low income in nonmetropolitan areas, where hearing loss is more prevalent.^5^ These longitudinal data provide an update to prior literature on hearing aid use and corroborate prior research on disparities in hearing care.^1,6^

Any association with financial and geographic access to hearing care may be explained by the resource-intensive process that accompanies hearing aid acquisition and long-term use. While initiatives such as the Over-the-Counter Hearing Aid Act of 2017 have focused on expanding access to hearing care, and others such as the Affordable Care Act and Medicaid expansion efforts may indirectly increase access, our findings highlight the need for more targeted approaches, given the lower reported use among low-income groups overall as well as a decrease in use among adults with low income who reside in nonmetropolitan areas. Notably, our data do not reflect the use of over-the-counter hearing aids, which were not available to the public until October 2022.

Our study is limited by a lack of audiometric data, which were added to NHATS in 2021. Furthermore, we did not consider device features or frequency of use. Last, while hearing loss is more prevalent in nonmetropolitan areas,^5^ the prevalence of hearing loss in metropolitan and nonmetropolitan areas should not change disproportionately over time. Future work should build upon this intersectional approach by investigating additional variables that may affect access to hearing care (eg, race and ethnicity or socioeconomic status) to gain a more comprehensive understanding of the factors that contribute to these disparities.

## Notes

Supplement.
Data Sharing Statement
