# Supplementary material for: Hearing Aid Use Trends Among Older Adults by Income and Metropolitan vs Nonmetropolitan Residence
Source: JAMA Netw Open. 2024 Sep 27;7(9):e2436140. doi: 10.1001/jamanetworkopen.2024.36140 (PMC11437379; doi:10.1001/jamanetworkopen.2024.36140)
Supplement: Supplement. — Data Sharing Statement [file jamanetwopen-e2436140-s001.pdf]

Data Sharing Statement

Bessen. Hearing Aid Use Trends Among Older Adults by Income and Metropolitan vs Nonmetropolitan Residence. *JAMA Netw Open*. Published September 27, 2024. doi:10.1001/jamanetworkopen.2024.36140

Data
Data available: Yes
Data types: Other (please specify)
Additional Information: The data that support the findings of this study are available from the corresponding author, SYB (sbessen2@jh.edu), upon reasonable request.
How to access data: The data that support the findings of this study are available from the corresponding author, SYB (sbessen2@jh.edu), upon reasonable request.
When available: With publication

Supporting Documents
Document types: None

Additional Information
Who can access the data: Researchers whose proposed use of the data has been approved
Types of analyses: For any reasonable purpose following review of request by authors
Mechanisms of data availability: After approval of a proposal or with investigator support
